# Evaluating Hemorrhage in Renal Cell Carcinoma Using Susceptibility Weighted Imaging

**DOI:** 10.1371/journal.pone.0057691

**Published:** 2013-02-25

**Authors:** Wei Xing, Xiaozhou He, Mohammad A. Kassir, Jie Chen, Jiule Ding, Jun Sun, Jiani Hu, Zishu Zhang, E. Mark Haacke, Yongming Dai

**Affiliations:** 1 Department of Radiology, Affiliated Third Hospital of Suzhou University, Changzhou, Jiangsu, China; 2 Department of Urology, Affiliated Third Hospital of Suzhou University, Changzhou, Jiangsu, China; 3 Department of Radiology, Wayne State University, Detroit, Michigan, United States of America; 4 Siemens Healthcare China, MR Collaboration NE Asia, Shanghai, China; University Clinic of Navarra, Spain

## Abstract

**Background:**

Intratumoral hemorrhage is a frequent occurrence in renal cell carcinoma and is an indicator of tumor subtype. We hypothesize that susceptibility weighted imaging (SWI) is sensitive to hemorrhage in renal cell carcinoma and can give a more diagnostic image when compared to conventional imaging techniques.

**Materials and Methods:**

A retrospective review of 32 patients with clear cell renal cell carcinoma was evaluated. All patients underwent magnetic resonance imaging (MRI) and 22 out of 32 patients also underwent a computed tomography (CT) scan. Hemorrhage was classified into 3 different categories according to shape and distribution. Histopathology was obtained from all masses by radical nephrectomy. The ability to detect the presence of hemorrhage using CT, non-contrast conventional MRI and SWI was evaluated, and the patterns of hemorrhage were compared.

**Results:**

Using pathologic results as the gold standard, the sensitivities of non-contrast conventional MRI, SWI and CT in detecting hemorrhage in clear cell renal cell carcinoma were 65.6%, 100% and 22.7%, respectively. Accuracy of non-contrast conventional MRI and SWI in evaluating hemorrhagic patterns were 31.3% and 100%, respectively.

**Conclusion:**

These results demonstrate that SWI can better reveal hemorrhage and characterize the pattern more accurately than either non-contrast conventional MRI or CT. This suggests that SWI is the technique of choice for detecting hemorrhagic lesions in patients with renal cancer.

## Introduction

Renal cell carcinoma (RCC) is the most common form of kidney cancer in adults. It accounts for approximately 3% of adult malignancies and 90% of neoplasms arising from the kidney [1], [2]. The 5-year survival rate can be as high as 95% for tumors that are less than 4 mm in size [3], [4] and confined to the renal parenchyma without venous invasion. The prognosis of patients with RCC correlates with tumor subtypes [5]. Intratumoral hemorrhage is an important indicator of RCC subtype. Hemorrhage is more common in clear cell RCCs (ccRCC) and collecting duct renal carcinomas than in papillary and chromophobe renal carcinomas [6]. Therefore, accurate detection of renal hemorrhage is of high clinical importance to the clinical management of patients with RCC.

Although renal masses could commonly be detected by ultrasonography and computed tomography (CT), magnetic resonance imaging (MRI) is particularly helpful in characterizing renal masses because of its advantage of providing excellent soft-tissue contrast [7]–[9]. Many MRI techniques have been developed to detect hemorrhage, including susceptibility weighted imaging (SWI). SWI is a gradient echo (GRE) method that combines the magnitude and phase information of the MR images to provide high sensitivity to susceptibility differences and/or changes, such as between hemorrhage and surrounding tissues [10]–[13]. SWI has been traditionally performed to enhance contrast between tissues with different susceptibilities in the brain using 3D acquisition, which has demonstrated superior sensitivity when compared to other imaging techniques in detecting lesions with microhemorrhage [12], [14], [15]. Technical barriers have prevented the use of 3D SWI in the abdomen. One example is breathing artifacts from long acquisition times. Recently, a new multi-breath-hold two dimensional (2D) GRE based SWI has been developed (a work in progress sequence, [WIP#608], Siemens Healthcare). Its superiority in siderotic nodule detection over conventional MRI technique has been demonstrated [16], [17]. Applying SWI to study renal cancer, however, has not been reported yet.

We hypothesize that multi-breath-hold 2D SWI is sensitive to hemorrhage in RCC and can give an accurate imaging appearance. In this retrospective study, we compared 2D SWI with non-contrast conventional MRI as well as CT in detecting the presence of hemorrhage in RCC and correlated the anatomic findings with pathologic findings.

## Materials and Methods

### Subjects

A retrospective review was performed of patients who underwent MR imaging for evaluation of renal masses during a 9-month period from March 2011 to November 2011. The retrospective study was approved by the Institutional Review Board of Affiliated Third Hospital of Suzhou University and was conducted in accordance with the Declaration of Helsinki. Written informed consent was obtained from all study subjects.

During the study period, a total of 43 consecutive patients with renal masses were available. 11 cases were excluded owing to angiomyolipoma (n = 5), papillary RCC (n = 4) and chromophobe adenoma (n = 2). Finally, the 32 patients (20 men and 12 women; range, 27–73 years; median age, 59 years) with ccRCC were included in our study.

### Imaging Examinations

All subjects were scanned at 3T (MAGNTEOM Verio, Siemens Healthcare, Erlangen, Germany) using a standard 12-channel phase array body-matrix coil. Twenty-two of them also underwent CT scanning before MRI examination.

CT examinations were performed on a 16-row MDCT scanner (Somatom Sensation 16; Siemens Medical Solutions) with 0.75×16 mm detector, 5 mm-thick slice, and technical factor of 120 kVp and 150 mAs. The CT protocol included imaging before and after administration of 100 mL of iodinated contrast medium (Iopromidol; Bayer Schering Pharma, Berlin, Germany), with 370 mg of iodine per milliliter. The scan range of CT covered from apex of right diaphragm to the lower pole of right kidney. After administration of contrast material, the first post-contrast scan started 30 seconds after the beginning of injection. The second post-contrast scan began after 70 s post contrast injection.

The protocol of MRI for all the patients was: (a) coronal breath-hold half acquisition single-shot turbo spin echo (HASTE) T2-weighted imaging (T2WI) (TR/TE, 800/91 ms; field of view, 380 mm×380 mm; matrix size, 117×256; slice thickness, 4 mm; gap 1.95 mm; flip angle, 160°; bandwidth, 781 Hz/pixel; generalized autocalibrating partially parallel acquisition [GRAPPA] acceleration factor, 2); (b) transversal GRE T1-weighted imaging (T1WI) (TR/TE, 161/2.5 ms; field of view, 285 mm × 380 mm; matrix size, 180×320; slice thickness, 5 mm; slice gap 1.0 mm; flip angle, 70°; bandwidth, 270 Hz/pixel; GRAPPA acceleration factor, 2); (c) transversal HASTE T2WI (TR/TE, 700/96 ms; field of view, 285 mm× 380 mm; matrix size, 168×320; slice thickness, 5 mm; gap 1.0 mm; flip angle, 150°; bandwidth, 488 Hz/pixel); and (d) transversal 2D breath-hold SWI (TR/TE, 162/10.3 ms; field of view, 285 mm × 380 mm; matrix size, 187×384; slice thickness, 5 mm; gap 1.0 mm; flip angle, 20°; bandwidth, 620 Hz/pixel; GRAPPA acceleration factor, 2). The total scan time of coronal T2WI, transversal T1WI and T2WI was 20 s, 33 s and 22 s, respectively. 2D SWI was performed through 2∼3 breath-holds, each lasted 12∼16 seconds including a 5 second break, and the total acquisition time was around 1 minute depending on the respiratory condition of the patient. Suspended respiration was most reproducible at end expiration with a brief coaching session prior to the examination.

The order of all transverse sequences performed was T2WI, SWI and then contrast enhanced T1WI and all slices were obtained in slice-match mode by choosing the option of ‘copy the reference’ on an operation workstation.

The 2D SWI post-processing was done inline and consisted of the following steps: 1) the *k*-space complex data of each channel from the 12 channel body-matrix coil were individually processed using a 32×32 homodyne high-pass filter; 2) the high-pass filtered complex images from each channel were weighted by the coil sensitivity factor and were combined to a final complex image, as described by the adaptive combine method [17], [18], and the final magnitude and corrected phase images were extracted; and 3) a normalized phase mask was calculated directly from this corrected phase image and multiplied with the final magnitude image four times to produce the final SWI image [16].

### Image analysis

All images were analyzed by consensus of two genitourinary radiologists with more than 5 years' experience on a commercial workstation (Syngo, Siemens Healthcare, Erlangen, Germany). In ambiguous cases, both reviewers reached a consensus in their decision.

The readers were not blinded to the fact that patients had RCC but did not know the histopathology results. The observers evaluated CT, MRI and SWI at different time points in order to minimize bias. The interval between each two time points was 7 days.

Lesions were considered hemorrhage if they were characterized by the following reference standards: (a) in CT scan: hyper-attenuating areas within a tumor with a CT value range from 40∼70HU [19], [20]; (b) in non-contrast MRI: hyperintense in T1WI and hypointense or hyperintense in T2WI; or hypointense in both T1WI and T2WI [21], [22]; (c) in SWI and phase imaging: hypointense [13]. The MRI slice with the largest area of hemorrhage was used to evaluate each tumor.

Hemorrhagic lesions were classified into three types according to the shape and distribution revealed on imaging: (a) punctuate hemorrhage - the area of each hemorrhagic lesion was less than 0.30 cm^2^; (b) patchy hemorrhage - hemorrhagic lesion occupied 5∼60% area of the tumor and (c) extensive hemorrhage - hemorrhagic lesion occupied more than 60% area of the tumor. If both punctuate and patchy hemorrhagic lesions were seen in the tumor and the sum of their areas was more than 60% area of the tumor, the lesion was marked as one with extensive hemorrhage.

### Histopathologic analysis

Histopathology was obtained from all masses by radical nephrectomy. All tissue specimens were evaluated retrospectively by a pathologist with 10 years' experience. According to the Fuhrman criteria, all cases were classified into grades I to IV.

The resected tumors were cut along the slice with the largest area of hemorrhage indicated by SWI for gross examination. Several measures were taken to match the SWI slice with the pathologic slice as accurately as possible. First, only patients receiving radical nephrectomy were included in the study. Second, the angle between the inner edge of the kidney and the central line of the body as well as the distance between the MRI slice with the largest area of hemorrhage and the axial slice with the largest diameter of tumor were measured on coronal T2WI. Third, a ruler was placed in the middle of a rectangular platform. The resected kidney was obliquely placed on the platform with the same oblique angle between the inner edges of kidney and the ruler as previously determined on T2WI. Fourth, the surgically resected specimen was cut in half along the slice with the largest diameter of the tumor perpendicular to the ruler, and then the pathological slice was obtained based on the distance measured in step 2.

### Statistical analyses

Statistical analysis was performed using SPSS (version 17.0; SPSS, Chicago, IL, USA). A chi-squared test was performed to compare hemorrhagic detection between CT, non-contrast conventional MRI and SWI. The patterns of hemorrhage on non-contrast conventional MRI and SWI were also compared. A difference of P<0.05 was considered significant.

The results of T1&T2 MRI, SWI, and CT were compared for sensitivity in detecting hemorrhage as well as accuracy in characterizing the pattern of hemorrhage using the pathologic findings as the gold standard. Sensitivity was defined as the number of cases positive for hemorrhage on both imaging and gross pathology divided by the total number of cases positive for hemorrhage on gross pathology. Accuracy was defined as the number of cases with the same hemorrhagic pattern seen on imaging as that seen on gross pathology divided by the total number of cases. Sensitivity and accuracy of non-contrast conventional MRI and SWI were calculated for the overall results as well as for every cancer grade and pattern of hemorrhage. For CT, sensitivity was calculated for the overall results as well as for every cancer grade.

## Results

### Histopathologic results

On pathologic examination, hemorrhage was differentiated from kidney tissues based on color [23]. In the cut surface, normal kidney demonstrated a lighter outer cortex and darker medulla with a central pelvis. The parenchyma of ccRCC appeared yellow-orange. Fresh and old hemorrhage were red and brown, respectively. Fibrosis in the tumor was gray. All of the tumors (n = 32) showed hemorrhage on gross pathology. Of those 32 tumors, 23 had patchy hemorrhage pattern and 9 displayed extensive hemorrhage pattern in the cut surface.

### SWI, MRI and CT results

Patterns of tumor hemorrhage as observed in SWI, T1WI and T2WI, CT and gross pathology were based on the criteria listed in the methods section, and are summarized in Table 1.

**Table 1:** Demographic Data on Patients with ccRCC.

				Pattern of Hemorrhage			
Patient NO.	Sex	Age	Grade	T1WI and T2WI	SWI	CT	Gross Pathology
1	M	50	I	patchy	patchy	Yes	patchy
2	M	45	I	patchy	patchy	No	patchy
3	M	54	I	No	patchy	No	patchy
4	M	48	I	No	patchy	NA	patchy
5	M	29	I	punctuate	extensive	NA	extensive
6	F	60	I	No	patchy	NA	patchy
7	F	37	I	No	patchy	No	patchy
8	F	69	I	patchy	patchy	No	patchy
9	M	59	I	No	extensive	No	extensive
10	F	43	I	punctuate	patchy	NA	patchy
11	F	59	I	patchy	extensive	No	extensive
12	M	64	II	No	patchy	NA	patchy
13	M	69	II	patchy	extensive	NA	extensive
14	F	48	II	patchy	patchy	No	patchy
15	M	49	II	No	extensive	No	extensive
16	M	61	II	patchy	patchy	No	patchy
17	M	66	II	No	patchy	No	patchy
18	M	48	II	patchy	patchy	Yes	patchy
19	F	54	II	punctuate	patchy	No	patchy
20	F	65	II	patchy	patchy	No	patchy
21	M	57	II	No	patchy	No	patchy
22	F	58	II	punctuate	patchy	NA	patchy
23	F	49	II	No	patchy	NA	patchy
24	F	58	III	patchy	patchy	Yes	patchy
25	M	66	III	patchy	extensive	Yes	extensive
26	M	44	III	patchy	extensive	NA	extensive
27	M	77	III	No	extensive	No	extensive
28	F	55	III	patchy	patchy	NA	patchy
29	M	66	III	punctuate	patchy	No	patchy
30	M	58	IV	patchy	extensive	Yes	extensive
31	F	66	IV	punctuate	patchy	No	patchy
32	F	33	IV	patchy	patchy	No	patchy

Note. NA =  Not available, Yes =  hemorrhage, No =  no hemorrhage

Non-contrast conventional MRI revealed hemorrhage in 21 cases. Six tumors displayed punctuate hemorrhage while the other 15 showed patchy hemorrhage. Non-contrast conventional MRI findings agreed with pathologic findings in 10 cases. In the other 11 cases with hemorrhage, non-contrast conventional MRI displayed less hemorrhage than that found on pathologic examination. In the remaining 11 cases, non-contrast conventional MRI showed no features of hemorrhage. Overall, non-contrast conventional MRI had 65.6% sensitivity with 31.3% accuracy.

SWI revealed hemorrhage in all 32 cases with 23 cases showing patchy hemorrhage and 9 cases displaying extensive hemorrhage. The pattern of hemorrhage revealed by SWI was the same as that revealed by pathology in all cases. This provided 100% sensitivity as well as 100% accuracy for SWI.

On CT imaging, hemorrhage was seen in only five tumors out of 22 making its overall sensitivity 22.7%. The amount of hemorrhage seen on CT was significantly less than that seen on non-contrast conventional MRI or SWI (P<0.001).

Table 2 lists the results by cancer grade. The sensitivity of non-contrast conventional MRI in detecting hemorrhage was 54.5% (6 out of 11) for Grade I tumors, 58.3% (7/12) for Grade II tumors, 83.3% (5/6) for Grade III tumors, and 100% (3/3) for Grade IV tumors. The accuracy was around 30% for all tumor grades. For SWI, both sensitivity and accuracy were 100% for all tumor grades. For CT, sensitivity was 14.3% (1/7) for Grade I tumors, 12.5% (1/8) for Grade II tumors, 50.0% (2/4) for Grade III tumors, and 33.3% (1/3) for Grade IV tumors.

**Table 2:** Sensitivity and Accuracy by Cancer Grade.

Imaging Mode	Cancer Grade	n	Sensitivity (%)	Accuracy (%)*
T1&T2	I	11	54.5%	27.3%
SWI	I	11	100%	100%
CT	I	7	14.3%	NA
T1&T2	II	12	58.3%	33.3%
SWI	II	12	100%	100%
CT	II	8	12.5%	NA
T1&T2	III	6	83.3%	33.3%
SWI	III	6	100%	100%
CT	III	4	50%	NA
T1&T2	IV	3	100%	33.3%
SWI	IV	3	100%	100%
CT	IV	3	33.3%	NA
T1&T2	All	32	65.6%	31.3%
SWI	All	32	100%	100%
CT	All	22	22.7%	NA

Note. 1 NA =  Not available; 2 *Accuracy =  the number of patients with the same MRI hemorrhagic pattern as pathologic hemorrhagic pattern divided by the number of patients in the corresponding tumor grade

Table 3 lists the results of non-contrast conventional MRI and SWI by pattern of hemorrhage using pathology as the gold standard. A statistically significant difference in detecting hemorrhage pattern was present between non-contrast conventional MRI and SWI (P<0.001). Non-contrast conventional MRI had 65.2% sensitivity in detecting patchy hemorrhage and 66.7% sensitivity in detecting extensive hemorrhage. Its accuracy was 43.5% for patchy hemorrhage and 0% for extensive hemorrhage. SWI was more sensitive than non-contrast conventional MRI in distinguishing both patchy (*P*<0.001) and extensive hemorrhagic lesions (*P*<0.005). The sensitivity and accuracy of SWI were 100% for both patchy and extensive hemorrhage.

**Table 3:** Sensitivity and Accuracy by Pattern of Hemorrhage.

Hemorrhage	n	T1&T2 Sensitivity (%)	T1 & T2 Accuracy (%)*	SWI Sensitivity (%)	SWI Accuracy (%)*
Patchy	23	65.2%	43.5%	100%	100%
Extensive	9	66.7%	0%	100%	100%

Note. *Accuracy =  the number of patients with the same MRI hemorrhagic pattern as pathologic hemorrhagic pattern divided by pathologic results

**Figure 1:**
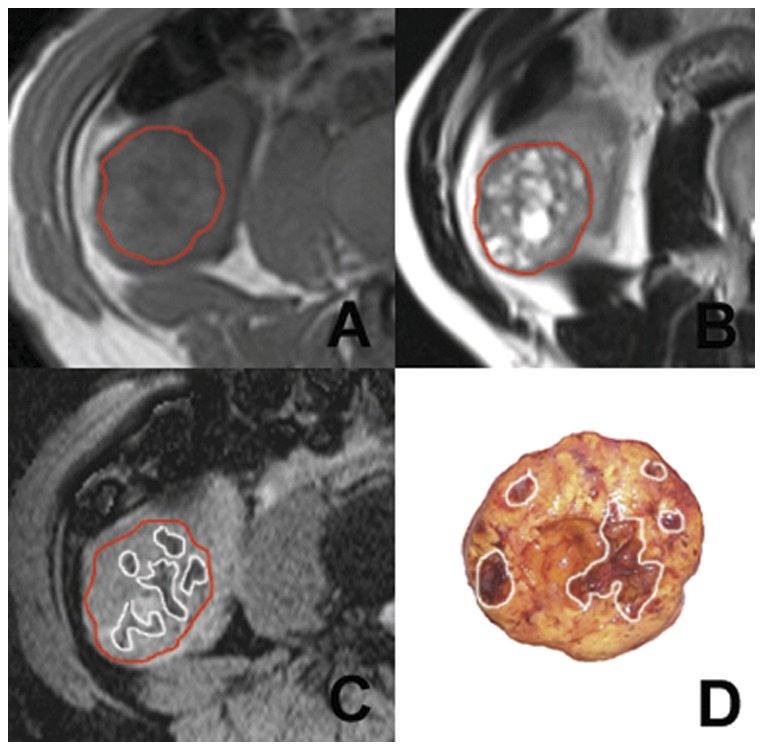
Right renal ccRCC with a grade II tumor in the Fuhrman system. The tumor appears multilocular (area demarcated by the red boundary). No marked hemorrhage is seen on either T1WI (A) or T2WI (B). On SWI (C), multiple patchy hemorrhagic lesions with hypointensity are present in the tumorous parenchyma (area demarcated by the white boundary). Good contrast between hemorrhage and the other tissues is present. In gross pathology (D), multiple patchy hemorrhagic lesions are seen in the tumor (areas demarcated by the white boundary).

**Figure 2:**
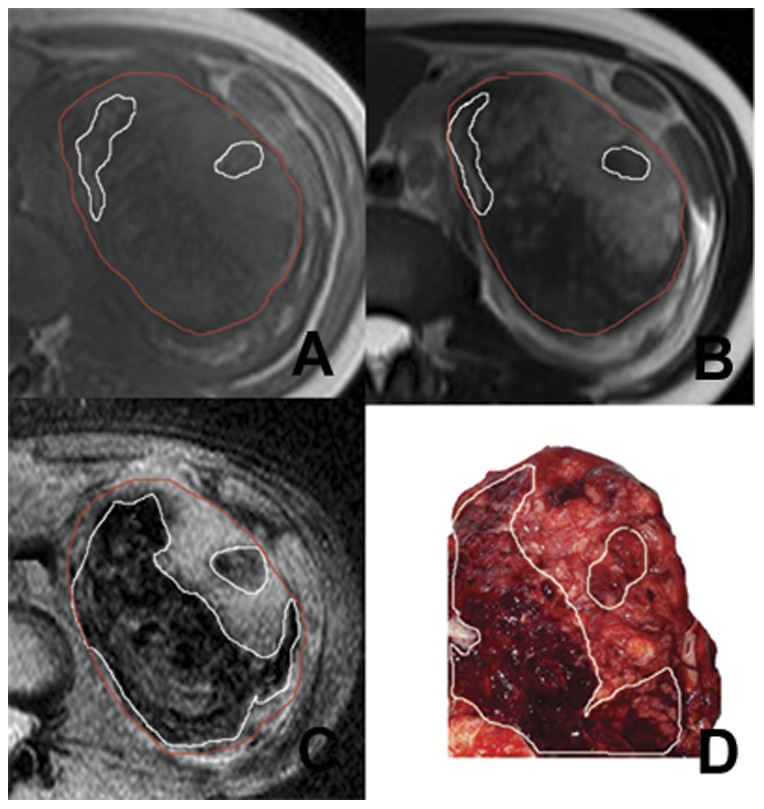
Left renal ccRCC with a grade III tumor in the Fuhrman system. Two patchy hemorrhage lesions with hyperintensity (area demarcated by the white boundary) are seen on T1WI (A) while one hypo- and one hyper-intensity hemorrhage lesions (area demarcated by the white boundary) are seen on T2WI (B) within the tumor region (area demarcated by the red boundary). On SWI (C), extensive hemorrhage lesions with hypointensity (areas demarcated by the white boundary) are present within the tumor region. In gross pathology (D), extensive hemorrhage lesions (areas demarcated by the white boundary) are seen in the tumor.

**Figure 3:**
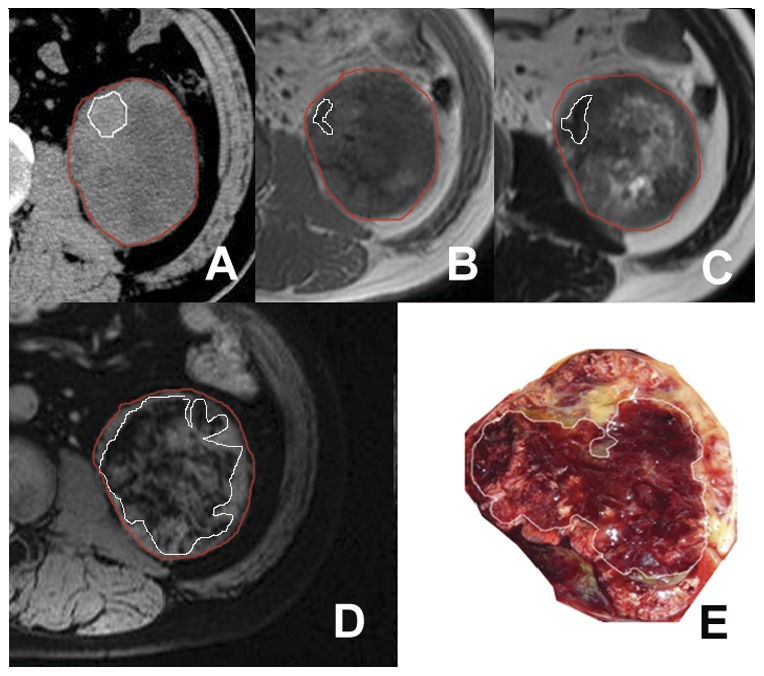
Left renal ccRCC with a grade III tumor in the Fuhrman system. In axial CT image, a patchy hyperattenuating area (area in white boundary) is seen in the tumor (area demarcated by the red boundary) (A). A hemorrhage lesion with hypointensity (area in white boundary) is seen on T1WI (B) and T2WI (C) within the tumor region (area demarcated by the red boundary). On SWI (D), an extensive hemorrhage lesion with hypointensity (area demarcated by the white boundary) is present in the tumor region. In gross pathology (E), an extensive hemorrhagic lesion (area demarcated by the white boundary) is seen in the tumor.

Figures 1 to 3 illustrate several typical cases, showing the difference in the visualization of intratumoral hemorrhage patterns between T1WI, T2WI, SWI, and gross pathology. In figure 1, no hemorrhage was seen on T1WI or T2WI, but patchy hemorrhage was seen on SWI and gross pathology. Figures 2 and 3 showed patchy hemorrhage on T1WI and T2WI. On SWI and gross pathology, the hemorrhage was classified as extensive. CT was performed for the patient in Fig 3. Patients in Figs 1 and 2 did not undergo CT scanning.

## Discussion

In this study, we retrospectively evaluated 32 patients with pathologically identified ccRCCs using CT, conventional T1WI and T2WI as well as a newly developed 2D abdominal SWI technique [16], [17]. We compared the results for sensitivity in detecting hemorrhage as well as accuracy in characterizing the pattern of hemorrhage using the gross pathologic findings as the gold standard. Our results demonstrated that abdominal SWI can detect hemorrhage better than non-contrast conventional MRI and CT. Also, non-contrast conventional MRI was better than CT. While the sensitivity of non-contrast conventional MRI in detecting hemorrhage in RCC and its accuracy in characterizing the hemorrhage pattern varied depending on different tumor grades and hemorrhage patterns, SWI demonstrated 100% sensitivity and 100% accuracy regardless of tumor grade or hemorrhage. This suggests that SWI has superiority over non-contrast conventional MRI in evaluating hemorrhagic lesions in ccRCCs. To our knowledge, this is the first SWI study on RCC, and the first systemic comparison between MRI hemorrhages with pathologic findings in the kidney [24]–[26]. Hemorrhage patterns by CT were not evaluated because CT images did not match MR images for all the patients.

The appearance of hemorrhage on MRI varies over time. The variation in signal intensity changes is related to the structure of the hemoglobin molecule as well as the hemoglobin degradation products present in the tissue [21]. Signal intensities of hemorrhage on MRI are influenced by the amount of hemorrhage, the hemoglobin degradation products present, the degree of red blood cell lysis, and the protein content. These factors make the signal intensity in hemorrhage varied and detection of hemorrhage difficult. In our study, non-contrast conventional MRI failed to detect hemorrhage in eleven patients. Also, the hemorrhagic lesions depicted on non-contrast conventional MRI were generally smaller than SWI.

MR images consist of two components: magnitude and phase. The phase is typically discarded in clinical routines because of artifacts and thus only magnitude images are analyzed for normal clinical MRI. But in SWI, the raw data is specially processed to reconstruct artifact-free phase images [27]. In this study, these artifact-free phase images provide important contrast information to differentiate hemorrhage from calcification as done in a previous study [28].

SWI has some important features that make it better than conventional MRI in detecting hemorrhage. As a hematoma ages, hemoglobin changes through several forms including oxyhemoglobin, deoxyhemoglobin, and methemoglobin. The magnetic susceptibility difference between oxygenated and deoxygenated hemoglobin, leads to a susceptibility difference between regions containing deoxygenated blood and surrounding tissues and results in signal-intensity cancellation. When the red blood cells are broken down into hemosiderin, hemosiderin in hemorrhagic lesions generates a strong susceptibility effect leading to phase changes and causing a T2* related signal decrease. These local susceptibility changes can be better evaluated by SWI because SWI enhances its contrast by jointly exploiting both magnitude and phase information. Theoretical investigations have indicated that a highly paramagnetic object measuring less than a quarter of a voxel can have a dramatic appearance within a single voxel, due to the ‘amplified blooming effects’ [29], this largely improves the sensitivity of SWI to hemorrhage. In contrast, conventional T1- or T2-weighted imaging is limited in the detection of hemorrhage due to their insensitivity to local susceptibility changes. As a matter of fact, evidence from various organs in the body have suggests that SWI is the best imaging method for detecting hemorrhage over ultrasound, CT or other MRI techniques [17], [29], [30]. For renal diseases, CT is known to be a better imaging modality than ultrasound in detecting hemorrhage [31], [32]. The result here clearly indicates that SWI is better than CT and non-contrast conventional MRI in detecting hemorrhage which is consistent with the literature [17], [29], [30]. This suggests that SWI should be the technique of choice to detect hemorrhagic lesions in patients with renal cancer.

The importance of accurately detecting hemorrhage has been discussed in many previous studies [5], [33]. Intratumoral hemorrhage in RCCs is helpful to distinguish different subtypes of RCC and can predict poor prognosis in patients with RCC [5], [33]. In a recent study, it was reported that hemorrhage and necrosis can be seen in 90% of renal tumors on gross pathology [34]. In addition, hemorrhage is a commonly pathological condition secondary to loco-regional therapies. Coagulative hemorrhagic necrosis in the treatment area can appear hyperintense on T1WI and complicate the interpretation of enhanced MRI [35]. Differentiating hemorrhage from the residual tumour is crucial for assessing the treatment response. The ability of SWI to detect hemorrhage with good sensitivity can provide valuable information to help diagnosis or therapeutic evaluation.

This study has some limitations. First, SWI images are prone to susceptibility artifacts produced by air within the gastrointestinal tract, which may have a similar imaging appearance to hemorrhage. Second, although all slices of the tested sequences were obtained in a slice-match mode, different kidney positions during each breath-hold will lead to a mismatch of slices and hence decrease the accuracy of the evaluation. This could be resolved when a registration technique is used. Third, our MRI reading was obtained by consensus for each pulse sequence. Determination of individual observer and inter-observer variability was not possible. Fourth, only ccRCCs with radical nephrectomy were evaluated. Fifth, ccRCCs with partial nephrectomy and other subtypes of RCCs were not included in this study. Sixth, because all patients showed intratumoral hemorrhage by SWI and histopathological examination in this study, the specificity of SWI was not established and should be assessed in future studies.

In conclusion, the newly developed multi-breath-hold 2D SWI used in this paper showed superiority over conventional T1WI and T2WI in detecting and evaluation of hemorrhagic lesions in RCC. SWI can be an important tool for imaging and characterizing hemorrhage in renal masses, thus playing an important role in clinical management of patients with RCC.
